# Current developments in soil ecotoxicology and the need for strengthening soil ecotoxicology in Europe: results of a stakeholder workshop

**DOI:** 10.1186/s12302-018-0180-y

**Published:** 2018-12-10

**Authors:** Janine W. Y. Wong, Bettina Hitzfeld, Michael Zimmermann, Inge Werner, Benoît J. D. Ferrari

**Affiliations:** 1 Ecotox Centre, Swiss Centre for Applied Ecotoxicology (Centre Ecotox Eawag-EPFL), Lausanne/Dübendorf, Switzerland; 2 Soil and Biotechnology Division, Federal Office for the Environment (FOEN), Bern, Switzerland; 3 Agro-environmental Systems and Nutrients Unit, Federal Office for Agriculture (FOAG), Bern, Switzerland

**Keywords:** Bioindicator, Monitoring, Biodiversity, Soil function, Ecosystem services, Soil fertility, Soil quality, Plant protection products

## Abstract

**Background:**

Soil is one of our most important resources and fulfills many ecological functions such as storage and filtration of water and nutrients, transformation of chemical compounds and nutrients, biomass production, and carbon storage. Such soil functions support ecosystem services provided by soils, which need to be protected to protect soil fertility. However, European soils often contain elevated concentrations of contaminants, putting biodiversity of soil organisms as well as the ecological functions and services at risk. To promote soil ecotoxicology in Switzerland, the Swiss Centre for Applied Ecotoxicology together with the Federal Office for Environment and the Federal Office for Agriculture organized a stakeholder workshop on 7 June 2018 with participants from research, governmental bodies, and associations. One goal of this workshop was to inform participants about currently available risk assessment approaches for soil, the soil risk assessment for plant protection products in Europe, available bioassays and bioindicators, and results of research projects on soil contaminants in Switzerland. Another goal was to discuss the needs for research in soil ecotoxicology in Switzerland and to identify next steps, potential projects, and future collaborations.

**Results:**

The main needs identified during the workshop were the establishment of (bio)indicators to measure soil fertility, functional parameters to determine soil functions, and the preservation of soil biodiversity. Another priority listed was the formation of a working group, which addresses the issue of the development of environmental quality standards for soil. The need for experimental field sites for implementing and testing new approaches or tools for assessing soil quality was also discussed.

**Conclusion:**

The next steps planned are two workshops with national and international experts in soil ecotoxicology to develop a soil monitoring concept for Switzerland and to find suitable bioindicators to evaluate soil fertility. Additionally, a literature review will be performed summarizing the current ecotoxicological state of the art with regard to the development of bioindicators in relation to the monitoring of plant protection products in Swiss soil, to evaluate their effects on soil fertility. Furthermore, all attendees agreed on the need for annual meetings or workshops where experts can present scientific results, participants can exchange information, and future projects and collaborations can be developed.

## Background

Numerous human activities lead to chemical contamination of soil, primarily via atmospheric deposition, infiltration of stormwater, as well as agricultural practices or improper waste disposal. In Switzerland, soils contain elevated concentrations of contaminants [1]. These can be transported into groundwater or taken up by crops and other plants, and thus be taken up by humans and animals via the food chain. Organism communities living in soil can be directly affected and put at risk which negatively affects diversity of soil-living organisms, soil fertility, and other soil characteristics. This, in turn, will reduce important ecological functions such as storage and filtration of water and nutrients, transformation of chemical compounds and nutrients, biomass production, and carbon storage. Such soil functions support ecosystem services provided by soils, which need to be protected to protect soil fertility.

Soil ecotoxicology is consistently gaining in importance as national and international awareness of the impacts of contaminants on soil communities, species diversity and associated ecosystem services is growing. In Europe, the Horizon 2020 Coordination and Support Action INSPIRATION (http://www.inspiration-h2020.eu) [2] was finalized in December 2017. The product of this project is the “European strategic research agenda (SRA) for Integrated Spatial Planning, Land Use and Soil Management” (http://www.inspiration-agenda.eu) [3]. The INSPIRATION SRA is based on research and innovation needs identified by more than 500 European stakeholders working as funders, scientists, policy makers, public administrators, and consultants. The SRA considers soil and land use management challenges, including the links between the soil-sediment and water system and topics such as health, energy, climate change, and resilient water supply. Furthermore, the European Commission and several European countries seek to put the 17 United Nations Sustainable Development Goals published in 2015 at the heart of their policy frameworks, priorities, and budgets. The SRA will be used to enable the Commission and individual countries to achieve these goals.

In Switzerland, the legal basis for the protection of soils exists since the entry into force of the Environmental Protection Act (“Umweltschutzgesetz”) in 1985. Healthy soils have been recognized also by the Swiss constitution as a fundamental requirement for a secure food supply (“Ernährungssicherheit”). This recognition is a driver for the planned establishment of a National Soil Competence Centre in Switzerland, which will be in charge of managing all available information on soils, and updating and implementing standards for the generation and interpretation of soil data in Switzerland. The National Action Plan for Plant Protection Products [4] finalized in 2017 includes measures for the protection of soils and soil organisms. It aims to reduce the environmental risks posed by plant protection products (PPP) by 50% and at promoting their sustainable use. One goal of the action plan is that residues of relevant PPP and their respective metabolites should be known and monitored from 2020 onwards. Another goal is that by 2027, the application of PPP that are persistent in soil (DT_50_ > 6 months) should be reduced by 30% compared to the period 2012–2015. It also calls for improving methods in retrospective risk assessment for terrestrial non-target species, and new approaches and indicators for monitoring soil fertility as threatened by chemicals.

## Objectives of the workshop

In this context, a first Swiss workshop on soil ecotoxicology took place in Duebendorf, Switzerland, on 7 June 2018. The workshop was organized and chaired by the Swiss Centre for Applied Ecotoxicology (Ecotox Centre), the Federal Office for the Environment (FOEN), and the Federal Office for Agriculture (FOAG). It had two goals: one was to inform the participants about the current developments in soil ecotoxicology in Europe (prospective and retrospective risk assessment of PPP in soil, the current state of soil risk assessment for plant protection products in Europe, available bioassays and bioindicators, and research projects on contaminants in Swiss soils). The second goal was to identify the needs for strengthening soil ecotoxicology in Switzerland and subsequently identify next steps, potential projects, and future collaborations. To attain these objectives, 39 regulators and representatives of agricultural institutions, federal offices and private industry as well as scientists mainly from Switzerland, but also from Germany and France, attended the workshop.

## Presentations

After a short welcome by the director of the Ecotox Centre, Inge Werner, a series of presentations were given by national and international experts as well as regulators from federal and cantonal agencies. Emmanuel Frossard (Swiss Federal Institute of Technology, ETH Zurich) provided a short summary on the national research project 68 (NRP 68 “Soil as a Resource”) [5] of the Swiss Science Foundation on the multifunctionality of soil. As mentioned before, soil fulfills a broad spectrum of functions and agricultural cultivation techniques should consider their potential adverse effects on these soil functions. He also stressed the need for the nationwide collection of soil information for reaching the goal of a more sustainable use of soil. Bettina Hitzfeld (FOEN) reported on future challenges regarding soil quality and which measures are required by the National Action Plan for Plant Protection Products. It aims at the reduction of the environmental risks posed by PPP and at promoting their sustainable use. Challenges for the execution of the Action Plan include (a) the lack of monitoring data and (b) the need for bioindicators to evaluate soil quality and fertility. Next, Katja Knauer (FOAG) gave an overview about the current risk assessment and risk management processes for PPP. She mentioned that data of chronic tests are primarily used in the risk assessment for soil organisms. Also, in the EFSA Scientific Opinion [6], it was recommended to adapt the predatory soil mite *Hypoaspis aculeifer* test to take into account the uptake of contaminated food and to develop a standardized test using isopods to include exposure via leaf litter. For microorganisms, it was proposed to advance the N-transformation test and to add a test using mycorrhizal fungi. In the presentation “Towards a better consideration of the effect of pesticides on soil organisms in the registration process of plant protection products in Europe”, Fabrice Martin-Laurent (Department of Agroecology, INRA, Dijon, France) highlighted the ecological importance of soil microorganisms and the protection of soil functions. Soil microorganisms, as all other soil fauna, are currently only considered in prospective PPP risk assessment. However, they are not considered in retrospective risk assessment in EU regulations, as the soil protection directive has not been adopted. There is an urgent need for standards to evaluate the effects of pesticides on microorganisms and their ecotoxicological consequences on soil functions. Sébastien Gassmann from the Geology, Soil and Waste Department of the Canton of Geneva presented a summary of measures taken for pesticide risk reduction in Geneva, where about 40% of the agricultural area is in close proximity of residential areas, which entails a large potential of conflict. A working group was formed to develop a PPP monitoring program, and to increase awareness among farmers regarding the use and potential effects of PPP. Daniel Wächter from the Swiss National Soil Monitoring program (Nationale Bodenbeobachtung; NABO) of Agroscope (Zurich, Switzerland) provided insights into data gained from a study on 90 PPP in Swiss agricultural soils. Residues of the majority of substances were detected in soil samples, even if the time of application was more than a year before the samples were taken.

The next block of presentations concentrated on current hazard and risk assessment approaches on soils. Jörg Römbke (ECT Oekotoxikologie GmbH, Flörsheim am Main, Germany) spoke about bioassays as part of the risk assessment process and the TRIAD approach [7], an integrative assessment method, which entails chemical analysis, ecotoxicological bioassays, and field studies. Since it is not possible to predict soil quality purely based on the concentration of contaminants in the soil, TRIAD offers a practical, validated and accepted approach to better estimate soil quality. Benjamin Pauget (TESORA, Arcueil, France) gave an introduction to the ‘Bioindicators’ program, a national research program implemented by ADEME (Agence de l’Environnement et de la Maîtrise de l’Énergie; the French Environmental and Management Agency) which covers monitoring, management concepts, and risk evaluation of soil. He presented multiple bioindicators such as the earthworm population index and the nematode index to the audience, which provide information on conditions and functions of soil and were already applied successfully in field studies. The Omega-3 index in plants or the sum of excess transfers (SET) index in snails can provide information about the transfer of contaminants. Another presentation on bioindicators was given by Sebastian Höss (ECOSSA, Starnberg, Germany). He presented tests with nematodes in mini-microcosms as useful bioindicator methods to measure soil quality and as a basis for risk assessments. Such mini-microcosms allow statistically sound testing of natural field communities under controlled laboratory conditions. The final talk of the session was given by Beat Frey (Swiss Federal Research Institute for Forest, Snow and Agriculture (Eidgenössische Forschungsanstalt für Wald, Schnee und Landschaft (WSL), Birmensdorf, Switzerland). He showed data from a study on mercury contamination of soils in the Canton Wallis. His data suggested that changes in the species community rather in the total number of species are a suitable way to evaluate the effects of soil contaminants.

## Outcomes and perspectives

In the afternoon, participants discussed two sets of questions regarding soil ecotoxicology in Switzerland. We first asked, “Where do you see needs in soil ecotoxicology? What do you expect from soil ecotoxicology?” We expected participants to come up with a list of the most important topics they think are necessary to advance in soil ecotoxicology. To enable the participants to reflect their own priorities and discuss the topics more thoroughly, we also asked them to prioritize the topics, especially to come up with a list of their top five priorities. Secondly, we asked, “Which actions should be implemented? Who should be responsible for each task?” Here participants were invited to develop ideas for the next steps and for future projects and collaborations. Furthermore, they were asked to think about institutions who should be in charge of the respective tasks.

Topics discussed encompassed a wide variety of points. The establishment of sound bioindicators to determine soil fertility/quality was determined as the top priority. This entails the need for a definition of soil fertility and quality. The second priority was the establishment of a parameter or a set of parameters to determine/measure the functional diversity of soil and the development of indicators. These indicators should preferably be easy to quantify, e.g., a simple gene analysis. The third priority was the preservation of soil biodiversity. Since our knowledge about species-specific functions of soil organisms is quite limited at this point, important species can be lost before their function in the soil has been identified. A large gene pool is more resilient to a variety of potential stress factors and thus supports the preservation of healthy soil functions. In addition to these top priorities, there were requests to form a working group, which should address the development of environmental quality standards (EQS) for chemicals in soils. Another important point was the need for experimental field sites for implementing and testing new approaches and tools for assessing soil quality. These sites could, e.g., be the sites NABO is currently monitoring for chemicals in Switzerland, but they do not have to be limited to them.

Another topic discussed was the importance of practical indicators for soil quality, which are sensitive to temporal variation and type of agricultural practice, as soil is not a homogenous matrix. Depending on soil type, pH, climate, soil use, etc. scenarios vary widely. Thus, to be able to have a common measure of soil quality, a range of reference values should be determined for each scenario. Measurements made in the field could then be compared to reference values of a corresponding scenario. Another need identified was to apply an effect analysis in monitoring programs. Changes in, e.g., biodiversity can thus be observed and measured. However, the cause(s) of such changes often remain unknown. To identify and evaluate the causes of biodiversity shifts is one of the future challenges in this area of research. For monitoring programs, it is important to take into consideration whether they are general (routine monitoring) or specific, e.g., focused on monitoring soil quality after treatment with PPP. Differences between the two were discussed. Monitoring requirements will also be different depending on the information available (application history) for a specific site.

Next steps: the NABO and the FOEN with support of the Ecotox Centre and EnviBioSoil (Lausanne, Switzerland) will hold two additional workshops with national and international experts from the field of soil ecotoxicology to develop a soil monitoring concept for Switzerland and to find suitable bioindicators to evaluate soil fertility. Additionally, EnviBioSoil will produce a literature review summarizing the current ecotoxicological state of the art with regard to the development of bioindicators in relation to the monitoring of PPP in Swiss soil, to evaluate their effects on soil fertility. Knowledge gained from the literature review will be integrated into the workshop process. Furthermore, all attendees agreed on the need for regular, e.g., annual meetings or workshops where experts can present scientific work, participants can exchange practical experiences, and future projects and collaborations can be developed.

## Abbreviations

ADEME: Agence de l’Environnement et de la Maîtrise de l’Énergie
EQS: Environmental Quality Standards
FOAG: Federal Office for Agriculture
FOEN: Federal Office for the Environment
NABO: Nationale Bodenbeobachtung
PPP: plant protection products
SET: sum of excess transfers
WSL: Eidgenössische Forschungsanstalt für Wald, Schnee und Landschaft
